# Human Dendritic Cells Express the Complement Receptor Immunoglobulin Which Regulates T Cell Responses

**DOI:** 10.3389/fimmu.2019.02892

**Published:** 2019-12-10

**Authors:** Usma Munawara, Khalida Perveen, Annabelle G. Small, Trishni Putty, Alex Quach, Nick N. Gorgani, Charles S. Hii, Catherine A. Abbott, Antonio Ferrante

**Affiliations:** ^1^Department of Immunopathology, SA Pathology at the Women's and Children's Hospital, North Adelaide, SA, Australia; ^2^College of Science and Engineering, Flinders University, Bedford Park, SA, Australia; ^3^School of Medicine, School of Biological Sciences and The Robinson Research Institute, University of Adelaide, Adelaide, SA, Australia

**Keywords:** dendritic cells, complement receptor immunoglobulin (CRIg), dexamethasone, cytokines, T cells, immunosuppression

## Abstract

The B7 family-related protein V-set and Ig containing 4 (VSIG4), also known as Z39Ig and Complement Immunoglobulin Receptor (CRIg), is the most recent of the complement receptors to be identified, with substantially distinct properties from the classical complement receptors. The receptor displays both phagocytosis–promoting and anti-inflammatory properties. The receptor has been reported to be exclusively expressed in macrophages. We now present evidence, that CRIg is also expressed in human monocyte-derived dendritic cells (MDDC), including on the cell surface, implicating its role in adaptive immunity. Three CRIg transcripts were detected and by Western blotting analysis both the known Long (L) and Short (S) forms were prominent but we also identified another form running between these two. Cytokines regulated the expression of CRIg on dendritic cells, leading to its up- or down regulation. Furthermore, the steroid dexamethasone markedly upregulated CRIg expression, and in co-culture experiments, the dexamethasone conditioned dendritic cells caused significant inhibition of the phytohemagglutinin-induced and alloantigen-induced T cell proliferation responses. In the alloantigen-induced response the production of IFNγ, TNF-α, IL-13, IL-4, and TGF-β1, were also significantly reduced in cultures with dexamethasone-treated DCs. Under these conditions dexamethasone conditioned DCs did not increase the percentage of regulatory T cells (Treg). Interestingly, this suppression could be overcome by the addition of an anti-CRIg monoclonal antibody to the cultures. Thus, CRIg expression may be a control point in dendritic cell function through which drugs and inflammatory mediators may exert their tolerogenic- or immunogenic-promoting effects on dendritic cells.

## Introduction

The Complement Receptor Immunoglobulin (CRIg), unlike other complement receptors, is expressed selectively in macrophages (1). The receptor plays a key role in the phagocytosis and clearance of bacteria in a complement dependent (1–3) and complement independent manner (4). But in addition, it has been reported to inhibit T cell responses. Vogt et al. (5) demonstrated that CRIg-Ig fusion protein inhibited the anti-CD3 or anti-CD3/28 antibody(s) induced mouse and human T cell proliferation and IL-2 production *in vitro*. When this fusion protein was injected into mice, there was a reduction in the numbers of antigen-induced CD8^+^ T cells and a reduction in the IFN-γ producing population. In these mice the Th-dependent IgG antibody response was reduced. CRIg expression in macrophages has been suggested to regulate the T cell response (6, 7). We demonstrate that human DC express CRIg mRNA and protein, including cell surface expression and that expression could be modulated by cytokines. Furthermore, dexamethasone was found to cause upregulation of CRIg expression on DC which inhibited the mitogen- and alloantigen-induced T cell response. This highlights an additional mechanism involved in the regulation of the adaptive immune response.

## Methods

### Cytokines and Cell Culture Reagents

Recombinant human cytokines used for DC treatments were as follows: LT-α (TNF-β), GM-CSF, M-CSF, IL-1β, IL-6, IL-4, TNF-α, IL-13, IFN-γ, IL-10 (ProSpec-Tany Technogene, Rehovot, Israel) and TGF-β1 (R&D Systems, Minneapolis, Minnesota, USA) were used in culture within a final concentration range of 5–80 ng/ml. Dexamethasone was used at a final concentration in culture at 30 ng/ml (Sigma-Aldrich, St. Louis, MO). All cell culture experiments utilized RPMI 1640 tissue culture medium, heat-inactivated (56°C/20 min) fetal calf serum (FCS), penicillin/streptomycin and L-glutamine (SAFC Biosciences, Lenexa, KS).

### Antibodies

Anti-human protein antibodies used in this study were as follows: mouse monoclonal anti-CRIg clone 3C9 (kindly provided by Dr. van Lookeren Campagne, Genentech, San Francisco, CA), phycoerythrin (PE)-conjugated and unconjugated anti-CRIg clone 6H8 (Santa Cruz Biotechnology, Dallas, TX, USA), fluorescein isothiocyanate (FITC)-conjugated anti-DC-SIGN/CD209 clone 120507 (R&D Systems). Isotype controls used were as follows: PE-conjugated and unconjugated mouse IgG_1_ (eBioscience, San Diego, CA), and FITC-conjugated mouse IgG_2b_ (R&D Systems). The secondary antibody used for Western blotting was horse-radish peroxidase (HRP)-conjugated rabbit anti-mouse IgG (Dako, Glostrup, Denmark).

### Preparation of Dendritic Cells

The study was approved by the CYWHS Human Ethics Committee (approval number REC 2165/4/2011). Dendritic cells were prepared from peripheral blood of healthy donors, who had given informed consent, utilizing previously described methods (8, 9). Blood was layered onto Ficoll® Paque PLUS (GE Healthcare, Uppsala, Sweden), *d* = 1.077, and centrifuged at 400 × *g* for 30 min. Firstly blood monocytes were prepared as described previously (9). The peripheral blood mononuclear cell (PBMC) layer was harvested and washed in RPMI-1640 medium with 2 mM L-glutamine, 100 U/ml penicillin, 100 μg/ml streptomycin and 10% heat-inactivated FCS. Then, the PBMCs were layered onto 46% iso-osmotic Percoll® gradient (GE Healthcare, Uppsala, Sweden) and centrifuged at 600 × *g* for 30 min. After centrifugation the lymphocytes were pelleted, and the upper monocyte-containing interphase layer was harvested and washed, with preparations routinely being of >98% viability and >90% purity by Giemsa. For all studies unless otherwise stated, monocytes were seeded at 1 × 10^6^ cells per 60 × 15 mm culture dish pre-treated with autologous plasma and left to adhere at 37°C for 1 h. Any contaminating non-adherent cells were removed, and the adherent monocytes cultured with RPMI-1640 medium with L-glutamine, penicillin, streptomycin, FCS, 50 ng/ml GM-CSF, and 20 ng/ml IL-4 at 37°C in an atmosphere of 95% air and 5% CO_2_ over 5 days for differentiation into DCs. The DCs were harvested by gentle pipetting and washed prior to use in experiments.

### DC-T Cell Co-cultures

Autologous DC and T cell co-cultures were setup using DCs as prepared above, with autologous T cells purified from the remaining lymphocyte fraction following the centrifugation of PBMC over 46% iso-osmotic Percoll® gradient. The T-cells were purified by subjecting the lymphocyte fraction through two cycles of nylon wool (Polysciences Inc., Warrington, PA) columns using an established protocol (10). The T-cell preparation was of >95% purity and >99% viability as determined by FACS analysis and trypan blue dye exclusion assay, respectively. The T cells were cryopreserved in liquid nitrogen until use (11). The DCs were added to 96-well round-bottom plate (Nunc) at 1 × 10^4^ cells/well and treated with dexamethasone for 24 h and washed. The cryopreserved T cells were thawed and added to the autologous DC (2 × 10^5^ T-cells/well). PHA was used as a stimulus in the appropriate wells (0.5 μg/well) (Remel Inc., San Diego, CA), with or without either anti-CRIg (clone 6H8) antibody or isotype control. The cells were cultured at 37°C in an atmosphere of 95% air and 5% CO_2_ for 72 h. Cells were pulsed with 1 μCi methyl-^3^H Thymidine (^3^H-TdR) (PerkinElmer, Waltham, MA) 6 h prior to harvest. ^3^H-TdR incorporation was measured as disintegrations per minute (DPM) in a Wallac 1409 liquid scintillation beta counter (Wallac, Turklo, Finland).

For allogeneic DC-T cell cultures, instead of autologous T cells, allogeneic T cells were isolated from fresh or cryopreserved PBMCs using the EasySep™ Human T Cell Isolation Kit (Stem Cell Technologies, Vancouver, Canada), and added to allogeneic DCs as the stimulus in a DC:T cell ratio of 1:10 as 2 × 10^5^ total cells/well, with or without anti-CRIg antibody or isotype control. DCs were untreated or dexamethasone treated DC at 2 × 10^4^ cells/well in 96-well round-bottom plates. Cells were cultured at 37°C in an atmosphere of 95% air and 5% CO_2_ for 120 h and pulsed with ^3^H-TdR 6 h prior to harvest. At harvest, culture supernatants were harvested and stored at −80°C for later quantification of cytokines, followed by measurement of the remaining cells for ^3^H-TdR incorporation. Cytokines in the culture supernatants were quantitated with BD™ Cytometric Bead Array kits for IFN-γ, TNF-α, IL-13, TGF-β1, IL-4, and IL-10 (BD Biosciences) following adaptation of the manufacturer's protocols for assay in 96-well v-bottom plates, with acquisition on a BD FACSCanto with an attached BD™ High Throughput Sampler (HTS), and analysis with FCAP Array v3 software (BD Biosciences).

In similar culture setups, we examined the T cells for the presence of Treg cells in the alloreactive stimulation as above. After 7 days of culture the cells were harvested and the levels of CD4^+^CD25^+^ CD127^lo^Foxp3^+^ cells measured by flow cytometry. Anti-human CD4-FITC, CD25-PE-Cy7, CD127-Alexa Fluor 647, Foxp3-PE, and corresponding isotype controls were from BD Biosciences. Cell surfaces were stained with appropriate antibodies for 20 min at room temperature (RT), washed once with PBS supplemented with 0.1% FCS, and incubation for 60 min at RT in Fixation/Permeabilization buffer (eBioscience). Following washing with Permeabilization buffer (eBioscience) and blocking with mouse IgG for 10 min at RT, intracellular staining of Foxp3 was performed in Permeabilization buffer with 30 min incubation at RT with the appropriate antibody or isotype control. Acquisition was performed on a BD FACSCanto and data analyzed using FlowJo v10.1 (FlowJo, LLC, Ashland, Oregon). The gating strategy to identify Foxp3^+^ Tregs is described in Supplementary Figure 3.

### Measurement of CRIg by RT-PCR and qPCR

For determination of total CRIg mRNA levels and isoform transcript detection, RNA was isolated using a RNeasy® Plus kit (Qiagen, Venlo, Limburg, Netherlands) according to the manufacturer's instructions, and treated with DNase I (DNA-free Kit, Ambion, Life Technologies, Mulgrave, Vic, Australia) to remove any genomic DNA contamination. The quantity of RNA was assessed on a NanoDrop™ (Thermo Fisher Scientific, MA, USA), and converted to cDNA using the iScript™ cDNA synthesis kit (Bio-Rad Laboratories, Hercules, CA).

Reverse transcriptase (RT)-PCR for CRIg isoform transcripts was conducted as previously described (12), using primers for isoform 1 (F1: TTTGTGGTCAAAGACTCCTCAAAGC; and R1: TGGCATGTGCCCTGGCT), isoform 2 (F2: TGTCCAGAAACACTCCTCAAAGCT; and R1), isoform 3 (F2; and R2: GAGAGACTTTCTTACCTGGCTGCTT), isoform 4 (F1 and R2), and isoform 5 (F1; and R3: GACACTTTGGGCTGGCTGCT). GAPDH primer sequences were used as previously described (12) (F: GAGTCAACGGATTTGGTCGT; R: GACAAGCTTCCCGTTCTCAGCCT). Separate reactions were set up for each isoform, containing 100 nM of each primer (pairing as described above), 1 μl of cDNA, and AmpliTaq Gold® 360 Master Mix (Applied Biosystems) in a 25 μl final volume. PCR reactions were performed with an initial denaturation at 95°C for 7 min, followed by 35 cycles of 95°C for 30 s, 60°C for 30 s, and 72°C for 60 s, and a final extension at 72°C for 7 min, using a SimpliAmp™ Thermal Cycler (Applied Biosystems). The RT-PCR products were visualized following electrophoresis on a 2% GelRed-stained agarose gel (Biotium) along with a 1 kb Plus DNA Ladder (Invitrogen).

qPCR for total CRIg mRNA expression was conducted as previously described (12) using the primer pair detecting all five known isoforms of CRIg (F: ACACTTATGGCCGTCCCAT; R: TGTACCAGCCACTTCACCAA) with the GAPDH primer pair described above for expression data normalization. Each reaction had a final volume of 20 μl containing 100 nM of each primer, 1 μl of cDNA, and iQ SYBR Green Supermix (Bio-Rad Laboratories). Triplicate reactions were assayed in an iQ5 Real Time Detection System with iQ5 Optical System v2.1 software (Bio-Rad Laboratories), with thermal cycling performed with an initial denaturation at 95°C for 5 min, followed by 40 cycles of 95°C for 30 s, 60°C for 30 s, and 72°C for 30 s.

### Measurement of CRIg Cell-Surface Expression

The expression of CRIg on the cell surface of DCs was measured by flow cytometry. At the conclusion of treatment, 1.5 × 10^5^ harvested DCs had Fc receptors on their surface blocked with ice-cold PBS supplemented with 0.5% (w/v) BSA, 10 mg/ml Intragam P, and 5% (v/v) human AB serum for 30 min. PE-conjugated anti-CRIg (clone 6H8) or isotype control antibodies, along with FITC-conjugated anti-CD209 antibodies were incubated with the DCs in a final staining volume of 50 μl for 30 min. The cells were washed in PBS with 0.5% (w/v) BSA, and following centrifugation (600 × *g* for 5 min), the cells were then fixed in PBS containing 1% (v/v) formaldehyde. A minimum of 20,000 events were acquired from the stained DC samples on a BD FACSCanto (BD Biosciences, CA, USA), with data analysis performed with FlowJo 10.1 (FlowJo, LLC, Ashland, Oregon). Doublets were excluded by gating with SSC-A vs. SSC-H. Trypan blue was used to determine cell viability (>95%) following harvest and prior to flow cytometric staining. Using 7-aminoactinomycin D (7-AAD), we were able to demonstrate specific DC viability in a set of replication experiments (Supplementary Figure 1) where similar results of enhanced CRIg expression by dexamethasone treatment was found.

### Western Blotting for CRIg Isoforms

Western blot for CRIg expression in DCs was performed using methods previously described (13). DCs harvested from each culture were lysed in 100 μl of 20 mM HEPES, pH 7.4, with 0.5% (v/v) Nonidet P-40, 100 mM NaCl, 1 mM EDTA, 2 mM Na_3_VO_4_, 2 mM dithiothreitol, 1 mM phenylmethylsulfonyl fluoride and 10 μg/ml leupeptin, aprotonin, pepstatin A, and benzamidine, for 2 h at 4°C with constant mixing. These samples were centrifuged at 12,000 × *g* for 5 min to obtain lysates (supernatants), and the protein content quantitated by Lowry assay, prior to the addition of Laemmli buffer supplemented with 3% β-mercaptoethanol. The lysates were boiled at 100°C for 5 min and 60 μg of protein loaded and electrophoresed on 12% SDS polyacrylamide gels, followed by transfer of protein onto nitrocellulose membrane (Pierce Biotechnology, Thermo Fisher Scientific, Rockford, IL). The membrane was stained with 0.1% Ponceau S (in 5% acetic acid) to ascertain protein loading equality. The amounts of the CRIg L, S and I isoforms were detected using monoclonal mouse anti-human CRIg clone 3C9 and HRP-conjugated rabbit anti-mouse IgG. The immune complexes on the membranes were visualized by enhanced chemiluminescence on a ChemiDoc XRS+ Imaging System and quantitated using Image Lab™ software version 3.0 (Bio-Rad Laboratories, Hercules, CA).

### Statistical Analysis

Statistical significance was calculated using GraphPad Prism 7.0 (GraphPad Software, Inc., La Jolla, CA, USA), with testing as follows: two-way ANOVA with *post-hoc* Bonferroni's Multiple Comparison testing for relative CRIg protein isoforms; Student *t*-testing for relative CRIg surface expression and dexamethasone-modulated CRIg mRNA expression; One-way ANOVA with *post-hoc* Dunnett's Multiple Comparison testing for cytokine dose-dependent CRIg mRNA expression; and One-way ANOVA with *post-hoc* Bonferroni's Multiple Comparison testing for ^3^H-TdR incorporation between DC-T cell co-culture treatments. Statistical significance was defined as *P* < 0.05.

## Results

### Expression of CRIg on Human DC

**Figure 1:**
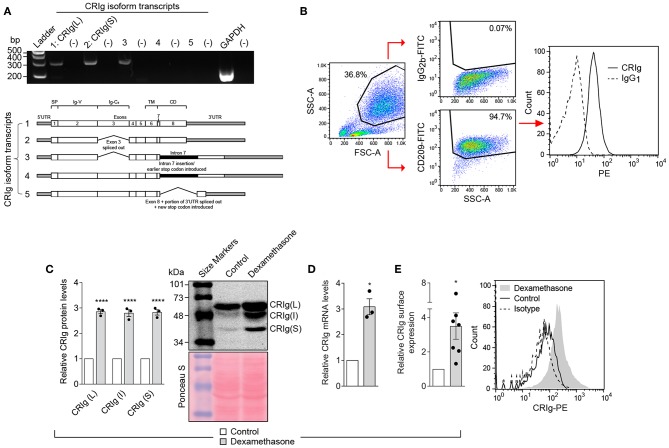
CRIg expression in MDDC and effects of dexamethasone. **(A)** CRIg isoform transcripts detected in DC. Agarose gel electrophoresis (2%) was used to visualize transcript variant amplicons generated from the cDNA of HMDC. Lanes are labeled 1, 2, 3, 4, and 5 representing each CRIg isoform with (–) to the right of each isoform lane indicating the respective no template controls. GAPDH was a positive control. Gels are representative of three experiments. **(B)** Gating strategy for CRIg expression on DC by flow cytometry. CD209^+^ cells were gated before assessing anti-CRIg antibody (6H8-PE) staining; representative histogram shown. **(C)** CRIg isoform expression by Western blot using anti-CRIg monoclonal antibody (clone 3C9). A representative blot of total protein stained with Ponceau S shows consistency of protein loading. Band intensity for each isoform (L, I, and S) over protein load was determined by densitometry. Data are expressed as fold-change over control DC (*n* = 3). **(D)** Relative CRIg mRNA expression as detected by qPCR, normalized to GAPDH, expressed as fold-change over control DCs (*n* = 3). **(E)** Relative CRIg cell-surface expression by flow cytometry. Data are expressed as fold-change in CRIg-PE (6H8) mean fluorescence intensity minus isotype control (IgG1) of treated over control DCs (*n* = 7); representative histogram shown. Data are presented as means ± SEM of experiments conducted with cells from different individuals. Significance levels are indicated by asterisks: *P < 0.05, ****P < 0.0001.

Human monocyte derived dendritic cells (MDDC) were generated in culture by treating monocytes with IL-4 and GM-CSF. The MDDC expressed CRIg mRNA by RT-PCR (Figure 1A) and CRIg protein on their surface by flow cytometry analysis (Figure 1B). Examination of transcripts showed that at least three isoforms of CRIg were present (Figure 1A). Furthermore, by Western blot analysis we identified the expression of the prominent long (L) and short (S) isoforms, as previously described in human macrophages (1) and an additional intermediate form migrating between the L and S isoforms (Figure 1C).

### Dexamethasone Increases CRIg Expression in DC Leading to Immunosuppression

Previously, we have demonstrated that the anti-inflammatory steroid dexamethasone is a strong enhancer of CRIg expression in human macrophages (12, 14, 15). In addition, it has been reported that DC generated under the influence of dexamethasone have a tolerogenic functional phenotype (16). It was therefore of interest to determine whether dexamethasone alters the expression of CRIg on DC. The MDDC were treated with varying concentrations of dexamethasone for 24 h, washed and CRIg expression measured. Dexamethasone caused an increase (3-fold) in CRIg mRNA levels (Figure 1D). This was reflected in an increase in CRIg protein measured by Western blotting (Figure 1C). Examination of the Western blots also revealed that dexamethasone caused an increase in the levels of all 3 isoforms of CRIg on DC (Figure 1C). The changes induced by dexamethasone were also evident in expression of CRIg on the surface of DC (Figure 1E), which has implications for the function of DC as antigen presenting cells and adaptive immunity.

**Figure 2:**
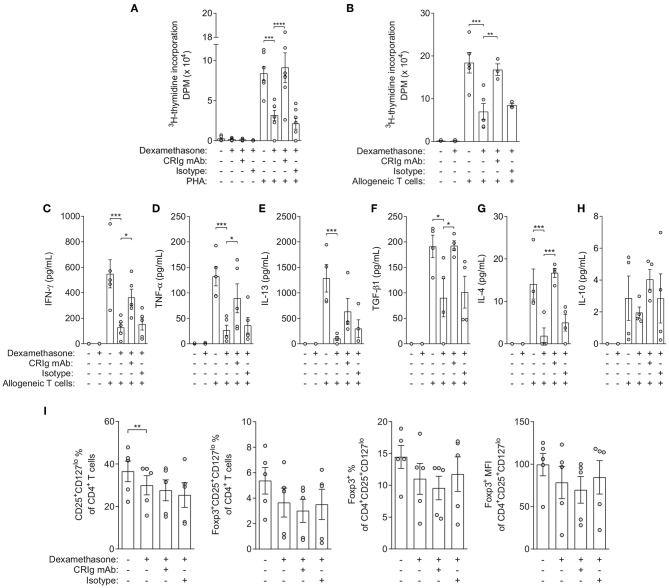
Effects of dexamethasone conditioned DCs and anti-CRIg monoclonal antibody on PHA- and allogeneic-induced T cell proliferation, cytokine production and generation of iTreg. **(A)** ^3^H-TdR incorporation (DPM) in autologous DC-T cell co-culture in the presence/absence of dexamethasone, anti-CRIg 6H8 or isotype control antibodies, and PHA. Data are presented as means ± SEM of 6 experiments conducted with cells from different individuals. **(B)** ^3^H-TdR incorporation (DPM) in allogeneic DC-T cell co-culture in the presence/absence of dexamethasone treated DCs, anti-CRIg 6H8 antibody or isotype control antibodies. Data are presented as mean ± SEM of 5 experiments. **(C–H)** Effects on cytokine production in allogeneic DC-T cell cultures. Data are presented as mean ± SEM of 4–5 experiments. **(I)** Effects on generation of iTreg in allogeneic DC-T cell cultures. Data are presented as mean ± SEM of 4 experiments. Note that normalized data for the above cytokine production and Treg generation is presented in Supplementary Figure 3. Significance levels are indicated by asterisks: *P < 0.05, **P < 0.01, ***P < 0.001, ****P < 0.0001.

To assess the functional consequences of increasing CRIg expression, we examined whether DC which had been treated with dexamethasone, expressing increased amounts of cell surface CRIg, were immunosuppressive in cell co-culture studies. Mononuclear leukocytes (MNL) from single individuals were separated into T cells and monocytes. The T cells were cryopreserved and the monocytes were treated with GM-CSF and IL-4 to allow development into DC. Then, the T cells were thawed and reconstituted with DC which had been pre-treated with either diluent or dexamethasone. The cells were stimulated with phytohemagglutinin (PHA) and proliferation was measured by a radiometric assay. The data showed that T cells cultured in the presence of dexamethasone conditioned DC were significantly depressed in proliferation (Figure 2A). Further studies examined the importance of surface expressed CRIg in the immunosuppression by adding anti-CRIg monoclonal antibody to the cultures (clone 6H8, Santa Cruz Biotechnology, Dallas, TX). The results showed that the suppression by dexamethasone conditioned DC could be completely prevented by the antibody (Figure 2A). The normalized data has been presented in Supplementary Figure 3.

In the second set of experiments, the effects of dexamethasone were assessed in an allogeneic T cell stimulation culture model. Monocyte derived DC were treated with dexamethasone, washed and added to allogeneic T cells. After 5 days of culture the culture fluids removed and the cells were replenished with fresh medium containing ^3^HTdR and harvested after 6 h of further incubation. The amount of radioactivity incorporated was determined and proliferation quantitated. The dexamethasone treated DCs caused a significant decrease in the allogeneic proliferative response (Figure 2B). When anti-CRIg monoclonal antibody was added to the cultures the effect was essentially abolished, suggesting that CRIg played a role in the immunosuppression. When we examined the cytokines IFNγ, TNF-α and IL-4, IL-13, IL-10, and TGF-β1 in the supernatants from these cultures, we observed that production of all these cytokines was significantly reduced in the presence of dexamethasone treated DCs except for IL-10 (Figures 2C–H), and that the addition of anti-CRIg monoclonal antibody prevented this suppression in cytokine production (Figures 2C–G). Examination of the lymphocyte population for Treg cells demonstrated that based on the expression of CD127, CD25, and FoxP3 expression there was no increase but if anything a decrease in this subset (Figure 2I). The normalized data has been presented in Supplementary Figure 3.

### Th1 and Th2 Cytokines Alter the Expression of CRIg

**Figure 3:**
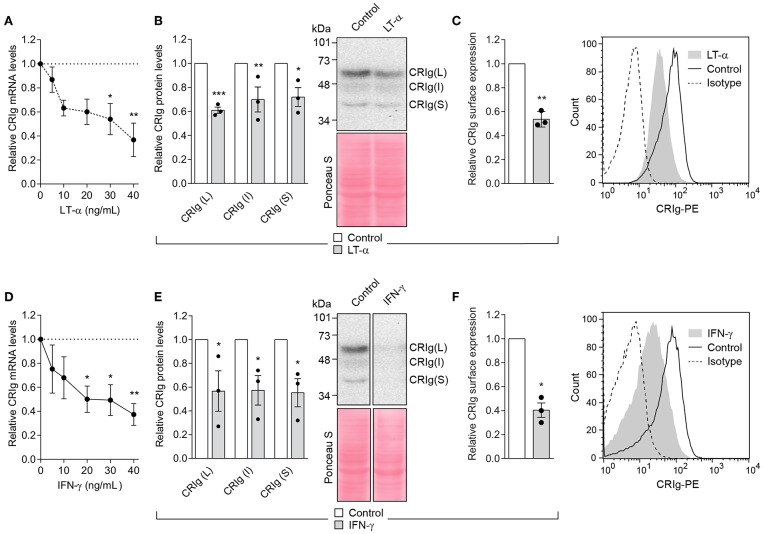
LT-α and IFN-γ decrease CRIg expression in DC. **(A,D)** DC were treated with varying concentrations of cytokines and the levels of CRIg mRNA determined after 24 h of culture. **(B,E)** In other experiments, CRIg isoform proteins were assessed by Western blot after cells were treated with 40 ng/ml of each of the cytokines. **(C,F)** The effects on cell surface expression of CRIg was examined after a similar treatment. Data are presented as means ± SEM of three experiments, each conducted with cells from different individuals. The blot image **(E)** was spliced to exclude intervening lanes that represent other treatments and the complete un-spliced blot can be found in Supplementary Figure 2. Significance levels are indicated by asterisks: *P < 0.05, **P < 0.01, ***P < 0.001.

In all of the following studies, we used CRIg^+^ DC that had been derived from monocytes cultured in the presence of GM-CSF and IL-4. No dexamethasone treatment was conducted. Our study examined the effects of four cytokines, IFN-γ and LT-α representing Th1 cell and IL-4 and IL-13 representing Th2 cell products on DC CRIg expression. When CRIg^+^ DC were treated with LT-α for 24 h, the cells showed a concentration dependent decrease in CRIg mRNA over a concentration range of 5–40 ng/ml (Figure 3A). This effect was supported by the finding that LT-α caused a significant decrease in total CRIg protein measured by Western blotting (Figure 3B), with a concomitant decrease of the L and S as well as the intermediate isoforms. A corresponding effect on cell surface expression was observed (Figure 3C). Treatment with 5–40 ng/ml of IFN-γ showed a similar decrease in CRIg mRNA, total protein and cell surface expression as seen with LT-α (Figures 3D–F).

**Figure 4:**
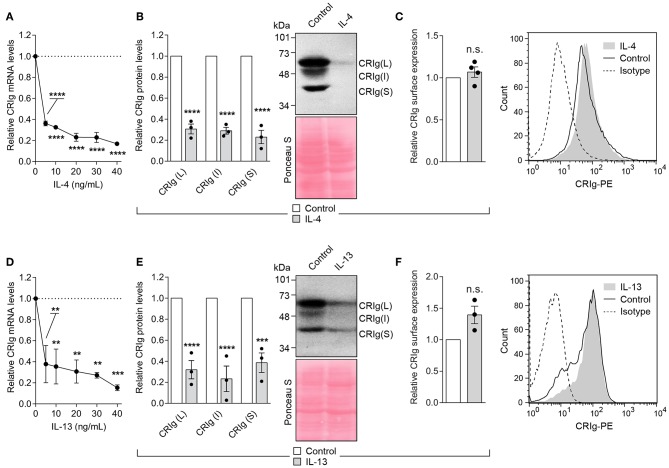
IL-4 and IL-13 down regulate CRIg expression in DC. **(A,D)** DC were treated with a dose range of the cytokines for 24 h and then examined for CRIg mRNA expression. **(B,E)** The levels of total CRIg isoform proteins measured by Western blot in DC treated with 20 ng/ml of cytokines. **(C,F)** Similarly treated DC were examined for surface expression of CRIg by flow cytometry. Data are presented as means ± SEM of at 3–4 experiments, each conducted with cells from a different individual. Significance levels are indicated by asterisks: **P < 0.01, ***P < 0.001, ****P < 0.0001, whilst n.s. indicates non-significance.

The Th2 cytokines, IL-4, and IL-13 caused an even more profound decrease in CRIg expression in the DC (Figure 4). The cytokines caused a decrease in CRIg mRNA expression over a concentration range of 5–40 ng/ml. A similar decrease was observed when total CRIg protein was measured by Western blot (Figures 4B,E). Expression of all three CRIg isoforms was decreased by treatment with either IL-4 or IL-13. However, this decrease was not reflected in a reduced expression of cell surface CRIg (Figures 4C,F).

### The Regulatory and Immunosuppressive Cytokines Increase CRIg Expression

**Figure 5:**
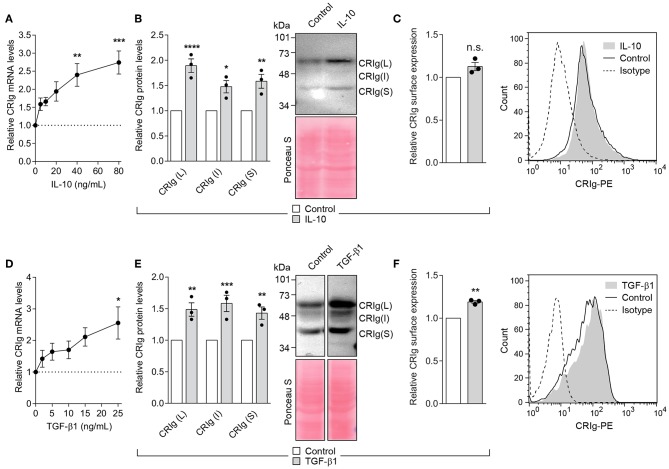
IL-10 and TGF-β1 increase CRIg expression in DC. **(A,D)** DC were treated with varying concentrations of the cytokines for 24 h and then examined for CRIg mRNA expression. DC were treated with either 40 ng/ml of IL-10 or 25 ng/ml of TGF-β1 and CRIg isoform protein expression in DC lysates **(B,E)** or CRIg expression on the cell surface **(C,F)** were measured. Data are presented as means ± SEM of three experiments, each conducted with cells from different individuals. The blot image in **(E)** was spliced to exclude intervening lanes that represent other treatments and the complete un-spliced blot can be found in Supplementary Figure 2. Significance levels are indicated by asterisks: *P < 0.05, **P < 0.01, ***P < 0.001, ****P < 0.0001, whilst n.s. indicates non-significance.

The cytokine TGF-β1 regulates inflammation and IL-10 has immunosuppressive activity (17–19). Their action could in part be through the regulation of CRIg on DC. We found that MDDC treated with IL-10 for 24 h showed a significant increase in CRIg mRNA expression in a concentration dependent manner (Figure 5A). Examination by Western blot showed a corresponding increase in CRIg protein expression (Figure 5B). However, this increase was not as evident in cell surface CRIg expression (Figure 5C). TGF-β1 also increased CRIg expression of mRNA, total CRIg protein and cell-surface expression on DC (Figures 5D–F). Examination of cell lysates subjected to Western blots showed that both TGF-β1 and IL-10 caused an increase in the levels of the L and S isoforms as well as the intermediate form on DC (Figures 5B,E).

### Effects of Pyrogenic Cytokines on the Expression of CRIg in DC

**Figure 6:**
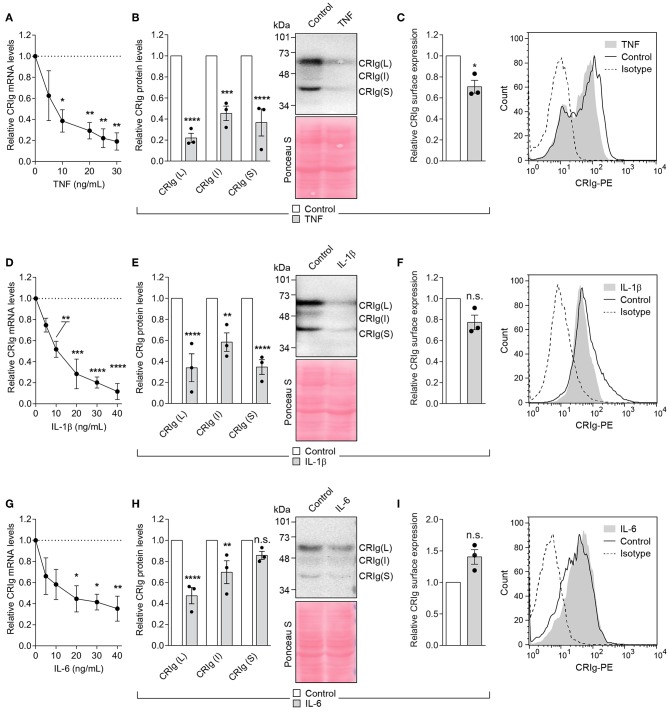
Effect of TNF-α, IL-1β, and IL-10 on CRIg expression in DC. **(A,D,G)** DC were treated with varying concentrations of the cytokines for 24 h and then examined for CRIg mRNA expression. For examination of CRIg isoform protein expression **(B,E,H)** and cell surface expression **(C,F,I)**, the cells were treated with 40 ng/ml of cytokine. Data are presented as means ± SEM of three experiments, each conducted with cells from different individuals. Significance levels are indicated by asterisks: *P < 0.05, **P < 0.01, ***P < 0.001, ****P < 0.0001, whilst n.s. indicates non-significance.

The cytokines TNF-α, IL-1β, and IL-6 are pyrogenic cytokines (20, 21) which have direct effects on monocytic cells, including the modulation of macrophage and DC differentiation (22–25) or macrophage function and cell death (26, 27). Since these are produced during the innate phase of the inflammatory response, they may influence the adaptive immune response through their effects on DC. It was therefore of interest to examine this group of cytokines on CRIg expression in DC. Cells treated for 24 h with either TNF-α, IL-1β, or IL-6 showed a significant decrease in CRIg mRNA expression, in a concentration dependent manner (Figure 6). This effect was reflected in the total CRIg protein expression decreased by the cytokines. However, there was no corresponding decrease in cell surface expression, apart from TNF-α (Figure 6). It was also evident that both TNF-α and IL-1β caused a decrease in all three isoforms of CRIg, shown by Western blot analyses but the S form was not significantly decreased by the IL-6 treatment (Figures 6B,E,H).

### Effect of Colony Stimulating Factors, M-CSF, and GM-CSF

**Figure 7:**
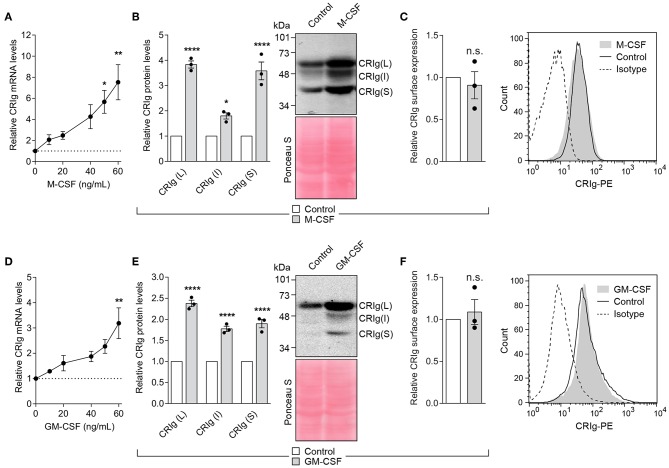
M-CSF and GM-CSF increase CRIg in DC. **(A,D)** DC were treated with varying concentrations of the CSF and the CRIg mRNA expression determined. Changes in CRIg isoform proteins **(B,E)** and cell surface **(C,F)** CRIg expression on DC treated with 40 ng/ml of each CSF are shown. Data are presented as means ± SEM of three experiments, each conducted with cells from different individuals. Significance levels are indicated by asterisks: *P < 0.05, **P < 0.01, ****P < 0.0001, whilst n.s. indicates non-significance.

Both M-CSF and GM-CSF have been reported to alter DC differentiation and/or function (22, 28). Examination of the effects of M-CSF and GM-CSF on the expression of CRIg in DC showed that cells treated with M-CSF display a marked increase in CRIg mRNA expression (Figure 7A). The increase paralleled the increase seen in total CRIg protein assayed by Western blot. The cytokine caused several-fold increase in the levels of CRIg protein expression (Figure 7B). Similar increases in CRIg expression of mRNA and total protein (Figures 7D,E) occurred in the presence of GM-CSF. However, we found that neither of these cytokines caused any changes in expression of cell surface CRIg (Figures 7C,F). As with other cytokines, all three isoforms of CRIg were concomitantly increased by the CSFs (Figures 7B,E).

## Discussion

The data provide evidence that CRIg is expressed by human monocyte derived dendritic cells (MDDC). Expression is observed at the mRNA, protein and cell surface level. The level of expression may dictate whether the cell promotes T cell responsiveness or unresponsiveness. Thus, increasing the surface expression of CRIg by treating with dexamethasone rendered the DC not capable of supporting the T cell response to PHA or alloantigen stimulation. Evidence that the dexamethasone-conditioned DC work through CRIg is provided by the finding that addition of an anti-CRIg monoclonal antibody to the cultures prevents the immunosuppression in both culture models. It has already been suggested that CRIg participates in adaptive immunity (5, 6, 29, 30). While Xu et al. (29) did not find CRIg expression in human MDDC, when these cells were transfected with the CRIg gene (representing the L form), the protein was expressed. The induced expression of CRIg in the transfected DC led to an immunosuppressed response or tolerance (29). This supports our data that CRIg expression regulates immune responsiveness. The inability to show expression in the non-transfected cells may be due to the fact that Xu et al. (29) treated the cells with TNF-α toward the end of their maturation phase. Our results show that TNF-α causes the down regulation of CRIg expression. While further studies need to be undertaken with DCs from different tissues, transcriptomic data indicate that CRIg is likely to be expressed in tissue DCs (Supplementary Figure 4). It is evident that expression ranges from medium to high in different DC types but expression can be as high as in macrophages. Of interest, although in limited studies, Tanaka et al. (31) described surface expression CRIg^+^ dendritic-like cells in the synovial tissue from rheumatoid arthritis, osteoarthritis and psoriatic arthritis patients.

Examination of the culture fluids in our DC-T allogeneic cell cultures for cytokine production supported the immunosuppressive effects of dexamethasone treated DCs, acting via CRIg expression. The dexamethasone-conditioned DCs-T cell cultures produced significantly less Th1 cytokines, IFN-γ, and TNF-α, as well as reduced Th2 cytokines, IL-4, and IL-13. In addition, the production of regulatory cytokine TGF-β1 was also reduced. With respect to all of these cytokines, the addition of anti-CRIg monoclonal antibody prevents the decrease in cytokine production. This indicates that the major effect precipitating the immunosuppression is the increased CRIg expression on the DCs. In mouse T cell cultures, Yuan et al. (7) showed that CRIg-Ig fusion protein suppressed the phosphorylation of signaling molecules, such as ZAP-70, ERK1/2, and Akt, thus acting early in the T cell activation response. Such inhibition was likely to cause the reported CRIg-Ig-mediated suppression of mTORC1 activation, thereby promoting inducible (i)Treg generation (32). Furthermore, the immunosuppressive effects of CRIg are unlikely to result from changes in proportions of Treg cells, since these were not increased but in fact decreased in these cultures, consistent with decreased production of the regulatory cytokine TGF-β1. In contrast, in mice CRIg-Ig fusion protein promoted the differentiation of Treg cells and the stabilization of Foxp3, although this was less evident when CRIg expressing macrophages were used (7). Whether these differences are due to macrophages vs. DCs or mouse vs. human leukocytes, as well as other factors, remain to be identified.

Expression on the cell surface indicates that CRIg will have the ability to mediate the tolerogenic properties of DC. Inevitably the level of CRIg expression on these cells may be a determining factor as to the potency of a resultant immunogenic or tolerogenic response the T cells may express, as the DC transform from providing an immunostimulatory signal to a tolerogenic signal following expression of CRIg (29). Thus, the composition of cytokine milieu at tissue sites is likely to be important in determining the role played by DC in the adaptive immune response, to which CRIg contributes. Our data demonstrate that cytokines significantly modulate the expression of CRIg in MDDC. CRIg expression on DC was increased by TGF-β1, IL-10, M-CSF, and GM-CSF. In comparison, LT-α, IFN-γ, IL-4, IL-13, TNF-α, IL-1β, and IL-6 decreased expression. In this manner, the cytokines could participate in tolerogenic vs. immunogenic responses, respectively through their ability to alter expression of CRIg on DC. However, it is not clear as to why DC treated with some cytokines did not show a corresponding alteration in expression at the cell surface. This may be an assay time related effect. But the ability of a cytokine to increase the intracellular CRIg levels may operate collaboratively with another cytokine to increase release to the cell surface, an area for future investigation. It is therefore tempting to speculate that inflammatory mediators may regulate expression at the transcriptional, translation and release to the cell surface. The regulatory effects of cytokines on CRIg expression has also been demonstrated for macrophages (12, 33). Supplementary Table 1 depicts the effects of cytokines on CRIg expression in MDM and MDDC. While most of the cytokines had similar effects on both cell types, LT-α, GM-CSF and the regulatory cytokines, IL-10 and TGF-β1, had the opposite effects on the two cell types.

The finding that IL-10 and TGF-β1 cause an increase in CRIg expression on DC is of interest and importance in adaptive immunity and immune responsiveness. Tolerogenic DC can be generated by immunosuppressive cytokines including IL-10, TGF-β1 (34–37), and immunomodulatory drugs, such as dexamethasone (38). Since tolerogenic DC are being considered as a therapeutic strategy in transplantation (39) and autoimmune inflammatory diseases (40, 41), these findings are likely to be helpful in developing tolerogenic DC for this purpose. IL-10 caused a substantial increase in CRIg mRNA and corresponding CRIg protein in human DCs. Dexamethasone treated DCs generates tolerogenic DCs that have reduced alloantigenic capacity, higher IL-10 secretion and inhibit Th2 differentiation of naïve CD4^+^ T cells in latex-allergic patients (42).

Our findings of CRIg being expressed by DC have important implications in autoimmunity, chronic inflammation and cancer (43). CRIg expression has been associated with decreased T cell and B cell responses (5, 44). The importance of CRIg in protecting against autoimmune inflammation has been demonstrated in experimental models of inflammatory arthritis (45), renal tubulointestitial injury (46), lupus nephritis (47), immune-mediated liver injury (48), type 1 diabetes (7, 30), and inflammatory bowel disease (49). In addition, the levels of CRIg expression in macrophages has been associated with disease severity in rheumatoid arthritis (31, 50) and patients with cirrhosis and ascites (51). In cancer, the level of CRIg expression by tumor associated macrophages has been shown to be a prognostic marker for tumors metastasizing, with high expression being prognostic for poor outcome (52–54). This also raises the potential for CRIg being a check point in the development of metastatic cancer and hence a drug target.

Our results demonstrate that three transcripts of CRIg are expressed in human MDDC. By Western blot analysis, we were able to identify the L and S isoforms along with an additional form not previously described and designated as the intermediate or I form. This most likely corresponds to the third transcript detected by PCR. However, because of lack of appropriate monoclonal antibodies specific for the different CRIg isoforms, we were not able to relate these to the changes seen in the Western blots. Nevertheless, it is evident that the I isoform is less prominent than the L and S isoforms. While the function of the I form remains unknown, it is tempting to speculate that since some of the extracellular domain is the same as the short form, its interaction with ligands should be the same. But because the I form has absence of intracellular phosphorylation sites as a result of alternative splicing, it is questionable that this form would be able to signal.

The ability of cytokines and dexamethasone to regulate the expression of CRIg was evident at the mRNA level and this correlated with protein expression, suggesting that inflammatory mediators and the immunosuppressive drug act at the pre-transcriptional level. It has been postulated by us that dexamethasone acts via the glucocorticoid receptor to downregulate CRIg expression as well as acting via the inhibition of PKCα activation and increasing CRIg expression in this manner (15). While the mechanisms of CRIg^+^ antigen presenting cell-induced immunosuppression remain to be elucidated, it has recently been demonstrated that engaging this receptor in macrophages reprograms the mitochondrial pyruvate metabolism and inhibits their activation (55). Here we have not only shown the expression of CRIg on DC, but that increased expression can lead to suppression of T lymphocyte proliferation. This provides important support for its role in protection against autoimmune inflammatory diseases and poor prognosis in metastasizing cancer (43). Furthermore, the findings expand our knowledge on CRIg and the regulation of the adaptive immune response, from that of its elegant role in clearance of pathogens and regulation of the alternative complement pathway activation (56, 57).

## Data Availability Statement

The datasets generated for this study are available on request to the corresponding author.

## Ethics Statement

This study was carried out in accordance with the recommendations of The Women's and Children's Hospital Network Human Ethics Committee with written informed consent from all subjects. All subjects gave written informed consent in accordance with the Declaration of Helsinki. The protocol was approved by the WCHN Human Ethics Committee, approval number REC 2165/4/2011.

## Author Contributions

UM performed the majority of the experiments and was involved in planning the experiments, collating the data, and writing the paper. KP was responsible for the allogeneic-induced T cell proliferation and cytokine production, as well as Treg measurements. AS assisted with the data presentation, writing of the manuscript, and conducting some experiments. TP conducted the cytokine measurements. AQ assisted with the collating of data, statistical analysis, and writing of the manuscript. NG critically read the manuscript and contributed its writing. CH was involved with reviewing of the data and writing of the manuscript. CA assisted with the planning of the research, supervised the work, and wrote the manuscript. AF initiated the study, supervised the research, reviewed the data, and wrote the manuscript.

### Conflict of Interest

The authors declare that the research was conducted in the absence of any commercial or financial relationships that could be construed as a potential conflict of interest.
