# Supplementary material for: Human Dendritic Cells Express the Complement Receptor Immunoglobulin Which Regulates T Cell Responses
Source: Front Immunol. 2019 Dec 10;10:2892. doi: 10.3389/fimmu.2019.02892 (PMC6914870; doi:10.3389/fimmu.2019.02892)
Supplement: Supplementary file 1 [file Data_Sheet_1.docx]

Supplementary Material

Human Dendritic Cells Express the Complement Receptor Immunoglobulin Which Regulates T Cell Responses

Usma Munawara^1,2,3,a^, Khalida Perveen^1,3^, Annabelle G. Small^1,3^, Trishni Putty^1,3^, Alex Quach^1,3^, Nick N. Gorgani^1,3,b^, Charles S. Hii^1,3^, Catherine A. Abbott^2^, and Antonio Ferrante^1,3*^

^1^Department of Immunopathology, SA Pathology at the Women’s and Children’s Hospital, North Adelaide, SA, Australia

^2^College of Science and Engineering, Flinders University, Bedford Park; SA, Australia

^3^The Robinson Research Institute, School of Medicine and School of Biological Sciences, University of Adelaide, Adelaide, SA, Australia

*** Correspondence:**Professor Antonio Ferrante
antonio.ferrante@adelaide.edu.au

**Supplementary Table 1.** The arrows indicate the effects of treating MDM and MDDC for 24 hours with the indicated cytokine/agent. MDM data from Munawara et al (2017).

| **Cytokine** | **MDM** | **DC** |
| --- | --- | --- |
| LTα | 🡩 | 🡫 |
| IFN-γ | 🡫 | 🡫 |
| IL-4 | 🡫 | 🡫 |
| IL-13 | 🡫 | 🡫 |
| IL-10 | 🡫 | 🡩 |
| TGF-β1 | 🡫 | 🡩 |
| TNF-α | 🡫 | 🡫 |
| IL-13 | 🡫 | 🡫 |
| IL-6 | 🡫 | 🡫 |
| M-CSF | 🡩 | 🡩 |
| GM-CSF | 🡫 | 🡩 |
| Dexamethasone | 🡩 | 🡩 |


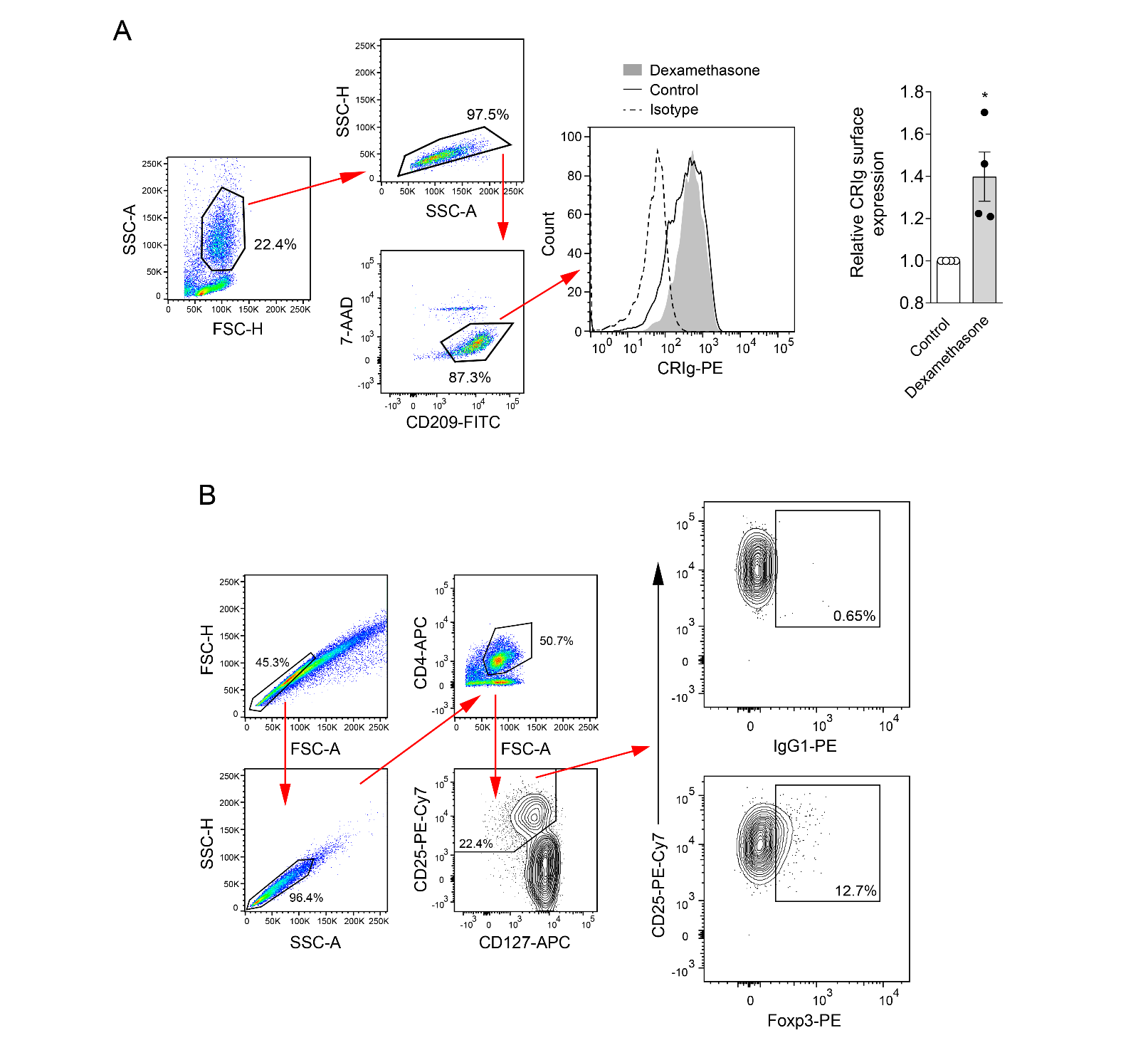


**Supplementary Figure 1.** Gating strategies for determining CRIg expression on the DC surface and Foxp3^+^ regulatory T cells in allogeneic DC-T cell co-cultures. **(A)** Representative plots show DC gating by capture of the high FSC/SSC population, followed by doublet exclusion by SSC-A vs SSC-H, and then exclusion of 7-aminoactinomycin D (7-AAD)^+^ cells (non-viable). Representative histogram overlays are also shown of isotype control and CRIg-PE staining in control and 24 h dexamethasone-treated viable DCs. The relative fold-increase in viable DC surface CRIg expression with dexamethasone treatment was shown to be significant from experiments of DCs from four individual donors. *, *P* < 0.05 by two-tailed t-testing. **(B)** Representative plots show Treg gating by capture of FSC and SSC singlets, followed by CD4^+^ T cell gating, and resolution of the CD25^+^CD127^lo^ Treg cell population. An isotype control (IgG1-PE) was used to adjudicate Foxp3^+^ Treg cells.


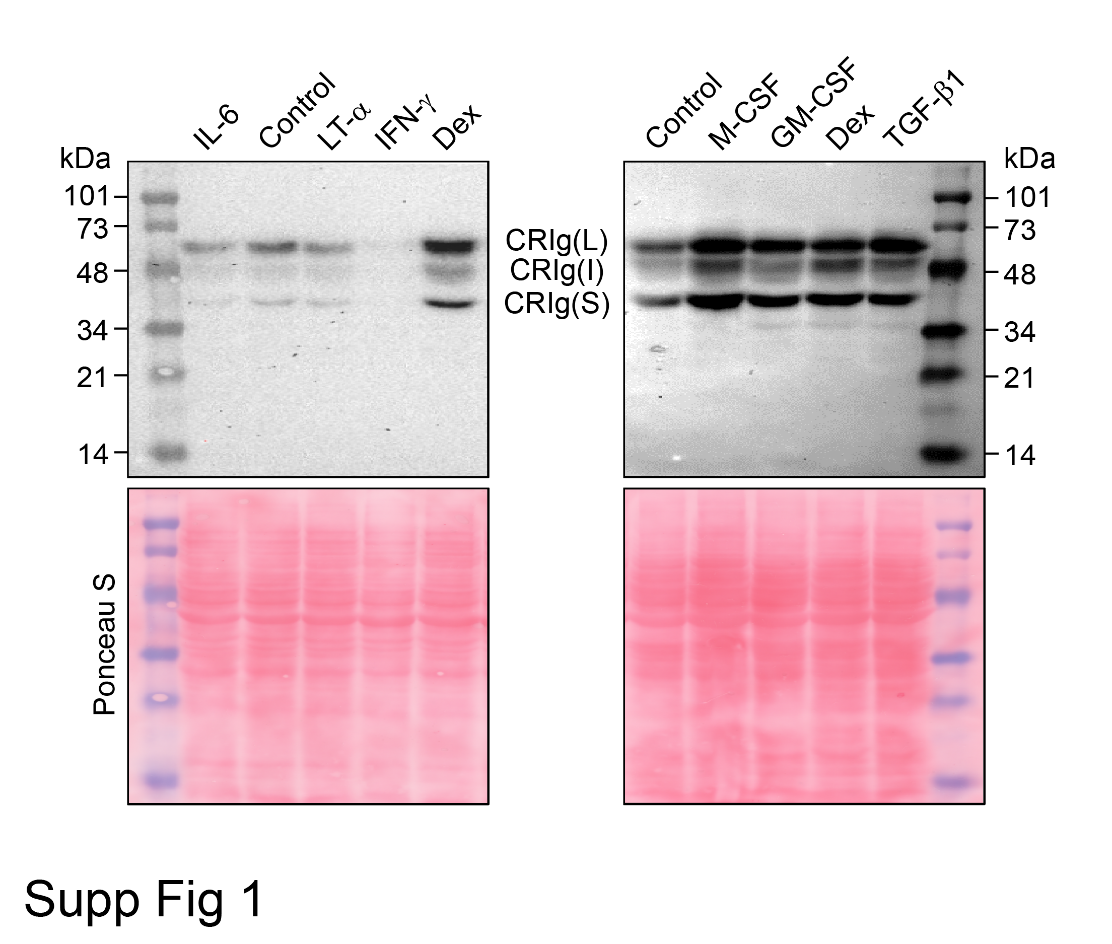


**Supplementary Figure 2.** Complete Western blots of CRIg isoforms in cytokine-treated dendritic cells from which spliced blots presented in Figure 3 and 5 were obtained. The left blot was the source of the spliced IFN-γ and corresponding control blot in Figure 3, whilst the right blot was the source of the spliced TGF-β1 and corresponding control blot in Figure 5. Each blot presents lysates from DC cultured in the presence of the indicated cytokines in an individual, that were examined by staining with CRIg 3C9 monoclonal antibodies. The corresponding Ponceau S staining shows the consistency of protein load. Low Range Prestained SDS-PAGE Standards (Bio-Rad Laboratories) were used for determining the long (L), intermediate (I) and short (S) forms of CRIg with ladder band sizes indicated in kilodaltons (kDa).


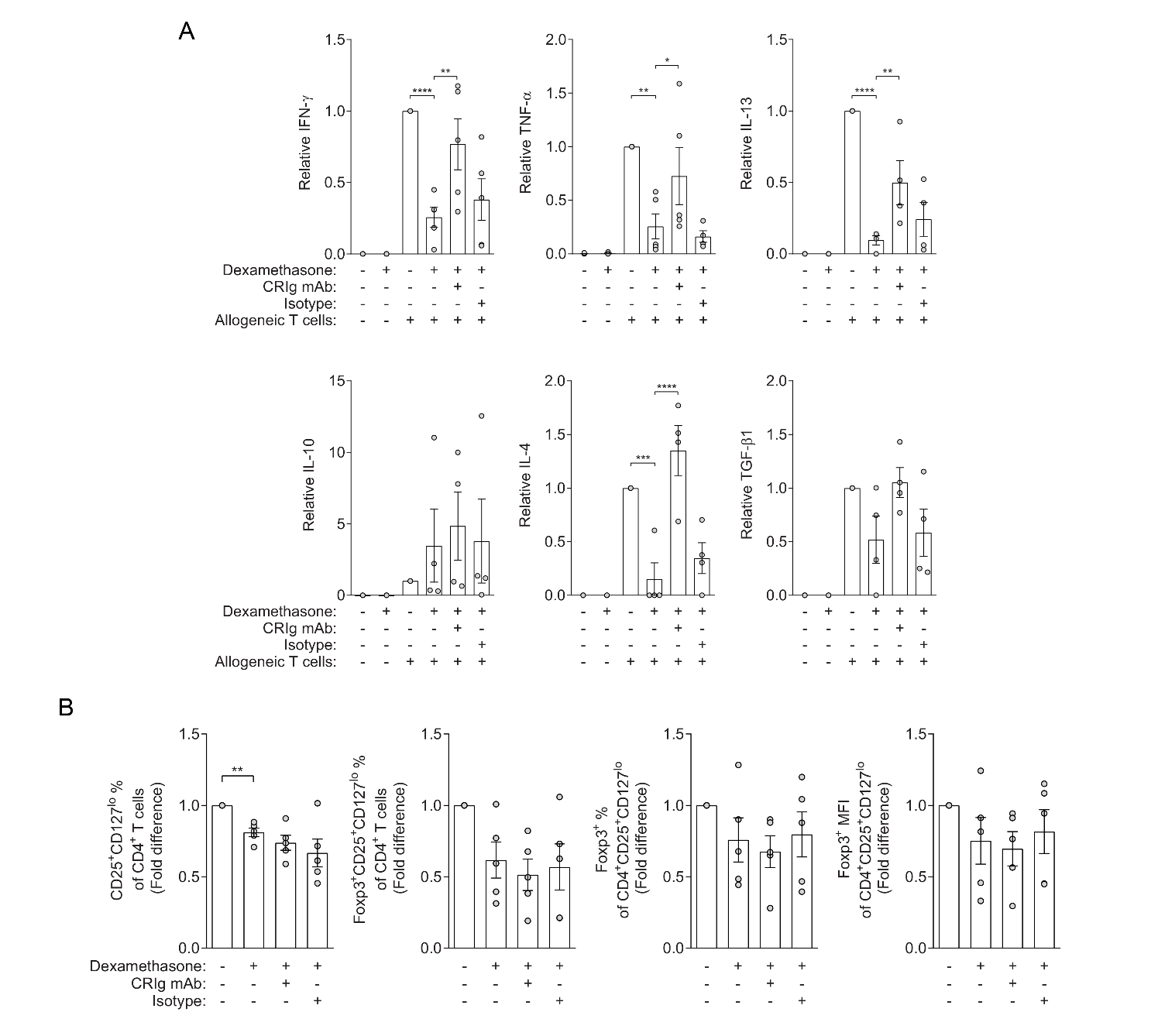


**Supplementary Figure 3.** Cytokine production and Treg cell populations in allogeneic DC-T cell-anti-CRIg reactions relative to the absence of dexamethasone treatment. **(A)** Relative IFN-γ, TNF-α, IL-13, IL-10, IL-4, and TGF-β1 production. **(B)** Relative CD25^+^CD127^lo^ and Foxp3^+^CD25^+^CD127^lo^ Treg percentages of CD4^+^ T cells, and Foxp3^+^ percentages and MFI of CD25^+^CD127^lo^ Treg cells. Significance levels are indicated by asterisks: *, *P* < 0.05, **, *P* < 0.01, ***, *P* < 0.001, ****, *P* < 0.0001.

**
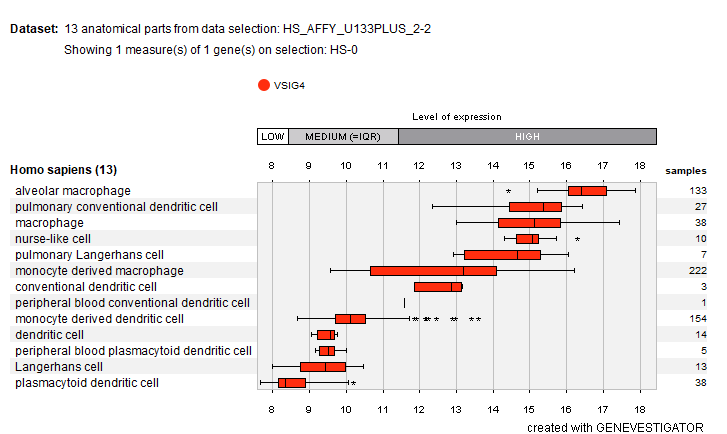
**

**Supplementary Figure 4.** *VISG4* expression levels in various human macrophages and dendritic cells. Data were extracted and compiled from public gene expression data repositories via Genevestigator V7.3.1 (Hruz et al. 2008). The expression levels are normalized by Genevestigator and presented as boxplots with the mean and interquartile range (IQR), and whiskers denoting 1.5 IQR from the lower and upper quartile, with asterisks indicating outliers. The sample sizes are indicated on each row of data.

**References**

Munawara, U., Small, A. G., Quach, A., Gorgani, N. N., Abbott, C. A., Ferrante, A. (2007) Cytokines regulate complement receptor immunoglobulin expression and phagocytosis of Candida albicans in human macrophages: A control point in anti-microbial immunity. *Sci Rep* 22;7(1):4050. doi: 10.1038/s41598-017-04325-0.

Hruz, T., Laule, O., Szabo, G., Wessendorp, F., Bleuler, S., Oertle, L., et al. (2008). Genevestigator v3: a reference expression database for the meta-analysis of transcriptomes. *Adv Bioinformatics* 2008, 420747. doi: 10.1155/2008/420747.
